# Non-Cytotoxic Agarose/Hydroxyapatite Composite Scaffolds for Drug Release

**DOI:** 10.3390/ijms20143565

**Published:** 2019-07-21

**Authors:** Markus Witzler, Patrick Frank Ottensmeyer, Martin Gericke, Thomas Heinze, Edda Tobiasch, Margit Schulze

**Affiliations:** 1 Department of Natural Sciences, Bonn-Rhein-Sieg University of Applied Sciences, von-Liebig-Str. 20, 53359 Rheinbach, Germany; 2 Institute of Organic Chemistry and Macromolecular Chemistry, Center of Excellence of Polysaccharide Research, Friedrich Schiller University of Jena, Humboldtstr. 10, 07743 Jena, Germany

**Keywords:** bone tissue engineering, agarose, hydroxyapatite, biocomposite, hydrogel, drug release

## Abstract

Healing of large bone defects requires implants or scaffolds that provide structural guidance for cell growth, differentiation, and vascularization. In the present work, an agarose-hydroxyapatite composite scaffold was developed that acts not only as a 3D matrix, but also as a release system. Hydroxyapatite (HA) was incorporated into the agarose gels in situ in various ratios by a simple procedure consisting of precipitation, cooling, washing, and drying. The resulting gels were characterized regarding composition, porosity, mechanical properties, and biocompatibility. A pure phase of carbonated HA was identified in the scaffolds, which had pore sizes of up to several hundred micrometers. Mechanical testing revealed elastic moduli of up to 2.8 MPa for lyophilized composites. MTT testing on Lw35human mesenchymal stem cells (hMSCs) and osteosarcoma MG-63 cells proved the biocompatibility of the scaffolds. Furthermore, scaffolds were loaded with model drug compounds for guided hMSC differentiation. Different release kinetic models were evaluated for adenosine 5′-triphosphate (ATP) and suramin, and data showed a sustained release behavior over four days.

## 1. Introduction

Bone infections, bone cancer, or major injuries can cause large bone defects of critical size [1,2]. In order to repair these large bone defects, bone-grafting materials such as autografts and allografts are used [3,4]. However, due to their limitations such as rejection rate, limited availability, and possible disease transmission, synthetic scaffolds are in the spotlight of current tissue engineering research [5].

Combining the scaffolds with human mesenchymal stem cells (hMSCs) that can be isolated from different sources such as bone marrow, umbilical cord, or fat tissue is of great interest. Since hMSCs originate from adult tissues, they do not cause severe ethical concerns such as embryonic stem cells. Contrary to pluripotent stem cells, hMSCs do not carry the risk of teratoma formation due to their limited plasticity [6]. Favorable for the use of hMSCs in bone reconstructive therapies is that the stem cells have the capacity to differentiate towards osteoblasts, which is the cell type mainly accountable for bone formation [7]. Moreover, it is possible to differentiate the hMSCs in vitro. This enables therapies where osteoblast precursors or the mature osteoblasts are differentiated on scaffolds. Since specific purinergic receptors are up- or down-regulated during osteogenic differentiation in vitro [8], signaling via these receptors can enhance osteogenesis [9]. The highly-conserved family of the purinergic receptors is divided into three subfamilies, the P1, P2X, and P2Y receptors. They use extracellular nucleotides as signaling molecules [10]. The four members of the P1 subfamily are G-protein coupled receptors that are activated by adenosine. The seven P2X receptors are ion-channels and are activated upon binding of ATP. The eight P2Y receptors are also G-protein coupled receptors and are activated by ATP, ADP, UTP, or UDP. Specific artificial ligands for these receptors are available and can be used to guide the cells into the osteogenic lineage [11].

Materials used for bone regeneration should be biocompatible, biodegradable, non-cytotoxic, and should enhance cell adhesion and proliferation. Most scaffolds presented in recent studies are made from either (bio-)polymers, (bio-)ceramics, or polymer/ceramic composites [1,5,12,13]. The latter combine positive effects from both types, while partly overcoming their respective disadvantages [14]. Natural bone itself is a composite of about 70% inorganic hydroxyapatite (HA) and 30% collagen type I, combining the stiffness of HA and the flexibility of collagen [15,16]. Calcium phosphates that resemble original bone cements, such as tricalcium phosphates (TCP), hydroxyapatite (HA), and octacalcium phosphate (OCP), are used as cements and pastes, but also as ceramic filler for polymer or hydrogel scaffolds. HA, the most stable calcium phosphate at body pH, is used in many applications since it promotes cell adhesion and proliferation [17,18]. However, it is brittle and has a low porosity. Micro- and macro-pores, which are crucial for successful vascularization, can be introduced via several different fabrication techniques such as 3D printing, electrospinning, salt leaching, or hydrogel formation [11,19,20].

Hydrogels are highly-hydrated 3D polymer structures that mimic natural extracellular matrix and their physico-chemical properties. Although scaffolds prepared from poly(vinyl alcohol), poly(acrylic acid), or natural polymers (e.g., collagen, chitosan, agarose) support fast tissue regeneration, they have low mechanical properties [21]. In contrast, polymer/ceramic composites have shown enhanced mechanical stiffness, cell adhesion, proliferation, and biodegradation [5,22]. Agarose, a natural polymer extracted from red algae, forms thermoreversible hydrogels. Upon cooling agarose solution below its setting temperature, agarose coils assemble themselves into helices, forming a porous polymeric network. Heating the gel above its melting temperature leads to a disassembly of the strands. This hysteresis loop may be repeated several times without changing the agarose’s properties [23,24]. Agarose is biocompatible and is studied intensively in the field of tissue engineering, e.g., for the regeneration of neural, cartilage, skin, and bone tissue [25]. For the latter, it is often used in hybrid composite systems, where the resulting scaffolds showed a high osteoconductivity [26,27] and induced new bone formation or osteoconduction [28,29].

Mineralizing agarose hydrogels also improved the mechanical properties and wettability of the scaffolds [30]. Aside from simply mixing HA powder with the agarose solution [31,32,33], there are several ways for in situ mineralization of HA inside the hydrogel that lead to more homogeneous scaffold materials. A common way is the wet alternate soaking process, where thin hydrogels are repeatedly placed in calcium- and phosphate-containing solutions for more than 10 cycles. This yields mineralized hydrogels, but is very time consuming due to the number of soaking and washing steps. Additionally, only thin gels can be homogeneously mineralized [26,29,34]. Another approach is the precipitation of HA inside an agarose solution containing phosphate by dropwise addition of calcium solution [30] or by the change of the pH value after hydrogel casting [35]. These techniques are less time consuming than alternate soaking, but may have more complex experimental setups, do not control the phase of calcium phosphate that is formed, or do not allow the easy incorporation of active ingredients.

Apart from giving structural guidance, the scaffold function is often combined with release properties for various kinds of bioactive ingredients such as antibiotics or growth factors and may even serve as a host compartment for cells. For example, ibuprofen and anti-osteoporosis drug zoledronic acid were released in vitro over three days from an agarose-hydroxyapatite scaffold and chitosan spheres [33]. Zoledronic acid (which has a high affinity to calcium phosphates) and recombinant human bone morphogenic protein-2 (rhBMP-2) were released in vitro and in vivo from a gelatin/CaSO_4_/hydroxyapatite system over four weeks [36]. rhBMP-2 was also used and was released in vitro from MSC-loaded collagen scaffolds and alginate/chitosan/hydroxyapatite scaffolds over 14 and 21 days, respectively [37]. The release behavior of the antibiotic amoxicillin from agarose-coated hydroxyapatite was investigated, as well, and a prolonged release of about three days was found [38]. However, only a few data on release kinetics were reported for these bone scaffold systems.

The current research adds to the broad field of tissue engineering and drug delivery. We present a fast and easy approach for preparing agarose/hydroxyapatite composite scaffolds. In detail, the composite preparation process is studied regarding the influence on the scaffold morphology, porosity, and their correlation to swelling behavior and mechanical stability. The biocompatibility of the scaffolds and the drug release kinetics of two model drugs structurally similar to osteo-influencing ingredients are also investigated and evaluated via different kinetic models.

## 2. Results and Discussion

During preparation, HA immediately precipitated as soon as the alkaline phosphate solution was added to the calcium-containing agarose solution, resulting in a homogeneous slurry that was used for casting the gels. Pure agarose solution remained transparent, solidifying upon cooling. All cast gels were of a cylindrical shape and were cut into approximately 10 mm-high cylinders for most analyses. AG100HA0 hydrogels were translucent, while added HA led to opaque, white gels with AG33HA67 having a polymer/ceramic ratio close to the original bone composition.

### 2.1. Scaffold Characterization

#### 2.1.1. X-Ray Diffraction

**Figure 1:**
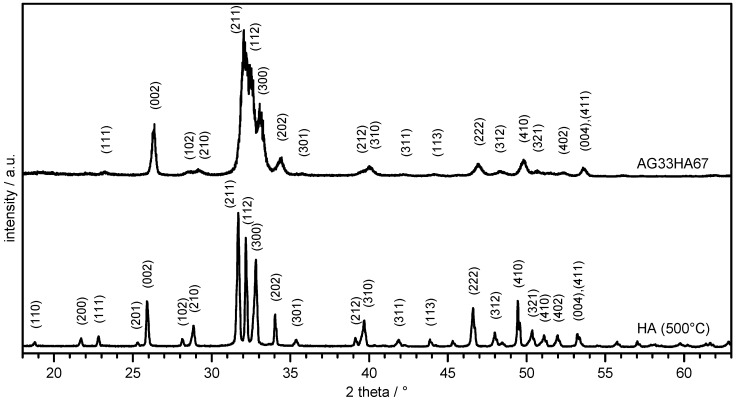
X-ray diffraction of hydroxyapatite (HA) dried at 500 ${}^{\circ }C$ and lyophilized agarose/HA composite (AG33HA67).

X-ray diffractograms of HA and AG33HA67 composite are shown in Figure 1. Reflexes of HA corresponded well to a (carbonated) HA phase (JCPDS PDF No. 09-432) and were indexed according to the literature [18]. The lyophilized scaffold showed much broader reflexes as it lacked thermal treatment; however, indexing of the major signals revealed also an HA phase.

Crystallite sizes of the (002) plane were 1.04 nm for pure hydroxyapatite and 0.55 nm in the composite. The degree of crystallinity was 92.1% and 37.8%, respectively. A small shift of the signals towards lower 2θ values was observable in the composite, indicating an influence of the agarose network on the crystallization of HA. This corresponds well to a previous study, where the agarose composite also showed shifted reflexes, and the degree of crystallinity was between 47 and 24%. The shifts in diffraction angle were due to the preferred crystal growth along the agarose gel network, which resulted in a slight distortion of the crystal lattice [30].

#### 2.1.2. Infrared Spectroscopy

**Figure 2:**
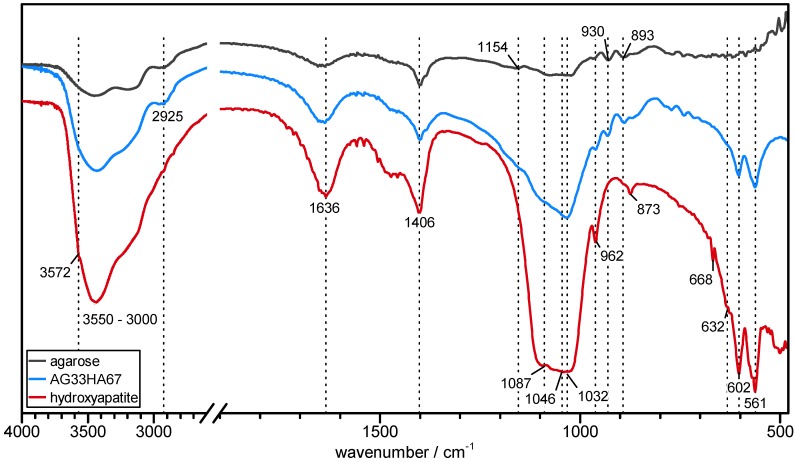
FT-IR spectra of agarose (black), hydroxyapatite (red), and agarose/hydroxyapatite composite (AG33HA67; blue).

FT-IR spectra of pure agarose, hydroxyapatite, and the composite are shown in Figure 2 and were used for the identification of the composite’s components. It clearly showed the main characteristic bands of both carbonated hydroxyapatite and agarose, confirming the presence of both. In detail, a broad OH stretching band between 3000 and 3600 cm^−1^ and a shoulder signal of $\nu_{S}$ free OH at 3572 cm^−1^ can be identified in all samples. Aliphatic C-H asymmetric stretching at 2925 cm^−1^ originating from the polysaccharide’s methylene groups can be found in agarose and the composite. OH deformation vibration at 1636 cm^−1^ may be overlapped by adsorbed bending water [39,40]. The signals at 1406 cm^−1^ and 893 cm^−1^ originated from in-plane O-H deformation coupled with C-H wagging in agarose and from $\nu_{3}$ CO_3_^2−^ in carbonated HA, respectively [39,40,41]. A weak signal at 1154 cm^−1^ from C-O-C acetal vibration in agarose was also observed as a shoulder in the composite [40]. In HA and the composite, there were very prominent phosphate triply-degenerated asymmetric P-O stretching *ν*_3*a*−*c*_ at at 1087, 1046, and 1032 cm^−1^ [18,41]. Weak CH_2_ twisting at 930 cm^−1^ originated from the agarose matrix [40]. The small signals at 873 and 668 cm^−1^ in the HA can be assigned to a $\nu_{2}$ and $\nu_{4}$ mode of carbonate, respectively [18,42]. Symmetric $n_{1}$ P-O stretching at 962 cm^−1^, $\nu_{4}$ O-P-O bending at 602 and 561 cm^−1^, and liberational mode $\nu_{L}$ of OH at 632 cm^−1^ [18,42] were also present in both HA and the composite. The absence of any other signals than those of agarose and (carbonated) HA led to the conclusion that the composite did not contain any other mineral phases or impurities.

#### 2.1.3. Porosity

BET measurements (Table 1) were performed to determine differences in the specific surface area of the samples, thus providing information on the respective microstructure. Lyophilization yielded a similar specific surface area $A_{sp}$ of 24–32 m^2^ g^−1^ for all samples regardless of composition, although $A_{sp}$ increased with the amount of added HA. These data concur with previously-published results, where similar results were found for lyophilized samples of mixed-in nano-HA in agarose solution before gelling [43] and microwave-assisted HA-AG composites [44]. Supercritical drying results in 3–6-times higher specific surface areas of 98–144 m^2^ g^−1^. Here, $A_{sp}$ decreased with the addition of more hydroxyapatite, with agarose gels having a significantly higher ($p<5\times 10^{-6}$ at the 95% level) specific surface area than both composite gels after supercritical drying.

**Figure 3:**
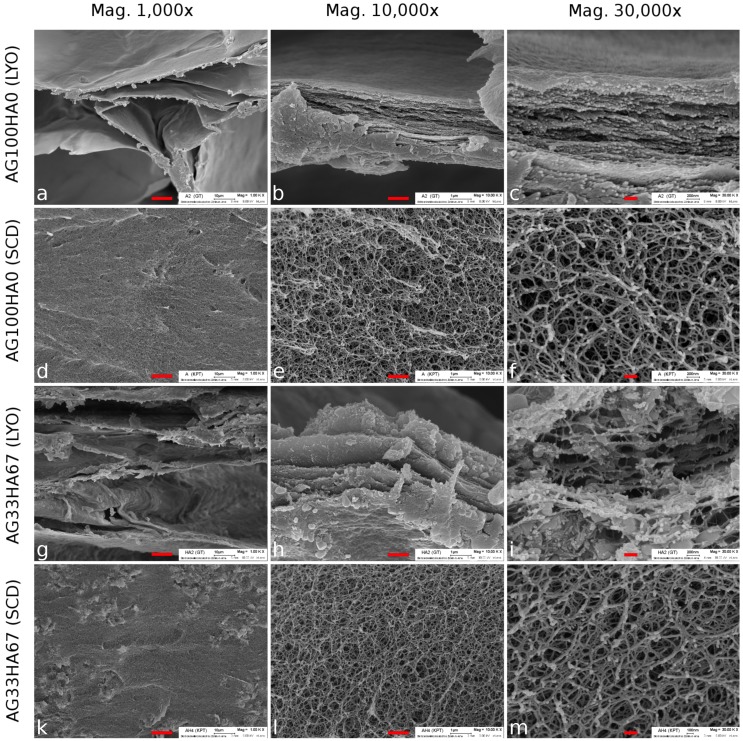
SEM images of agarose lyophilized (LYO; (**a**–**c**)) and supercritically-dried (SCD; (**d**–**f**)) and AG33HA67 composite LYO (**g**–**i**) and SCD (**k**–**m**) at three different magnifications. The scale bar is 10 μm (left), 1 μm (middle), and 0.2 μm (right), respectively.

**Figure A1:**
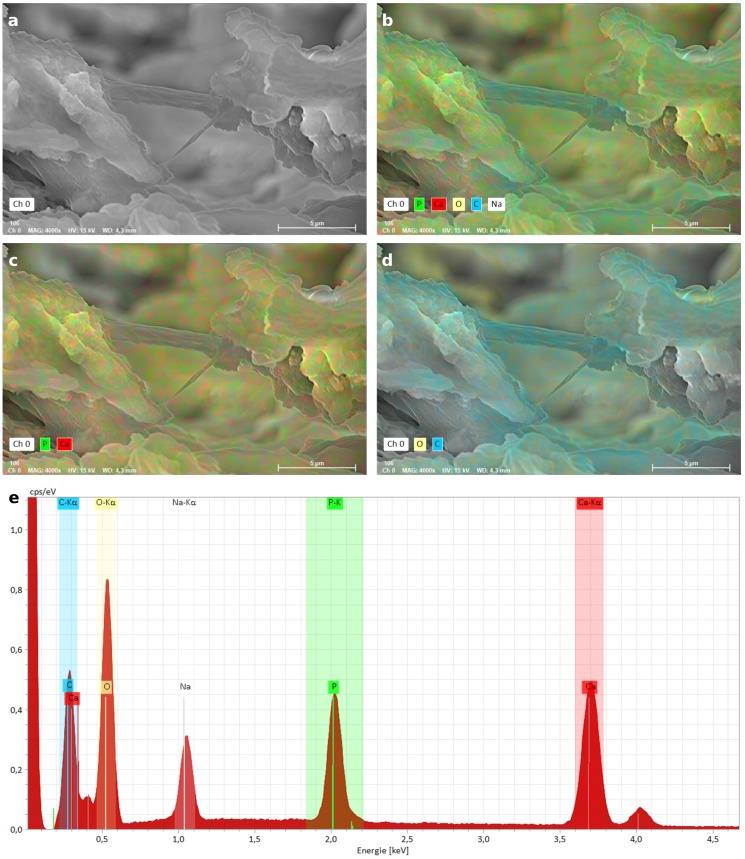
EDX mapping of a supercritically-dried AG33HA67 scaffold. (**a**) SEM picture; (**b**) mapping overlay of carbon (blue), calcium (red), phosphorus (green), oxygen (yellow), and sodium (white); (**c**) mapping overlay of calcium (red) and phosphorus (green); (**d**) mapping overlay of carbon (blue) and oxygen (yellow); (**e**) EDX spectrum of mapping data. The scale bar in mappings is 5 μm; voltage 15 kV; working distance 4.3 mm.

Scanning electron microscopy (SEM) was also used to evaluate the internal structure of the lyophilized (LYO) and supercritically-dried (SCD) gels and to determine pore sizes. Figure 3a–c shows LYO AG100HA0, and Figure 3d–f shows the SCD agarose sample. Figure 3g–i shows the LYO AG33HA67 composite and Figure 3k–m the SCD composite sample. At lower magnification, both lyophilized samples exhibited large pores in the range of several hundred micrometers (Figure 3a,g). This was due to the growth of ice crystals during the freezing process, forcing the agarose/composite to form layers. These layers or sheets (up to 2 μm in thickness) can be seen at higher magnifications (Figure 3b,c,h,i). In the composite, additional particles of HA were visible on and between the agarose sheets. These particles were between 150 and 500 nm in diameter (Figure 3h,i). In contrast to the lyophilized samples, the supercritically-dried ones did not exhibit large pores at lower magnifications, but seemed to be very homogeneous (Figure 3d,k). At higher magnifications, highly porous and very fine network structures (strand thickness about 35 nm) became visible, with no differences between the pure agarose and the composite. Interestingly, no distinct HA particles could be seen in the network structure, which suggests a coating of the agarose strands with hydroxyapatite. This was confirmed by EDX analysis (Figure A1), where a homogeneous distribution of carbon, calcium, and phosphorus was found throughout the scaffold.

The SEM observations matched the results measured by BET, which showed a much higher specific surface area for SCD samples. While the internal structure was destroyed during lyophilization, leading to larger pore sizes, supercritical drying preserved the fine hydrogel network structure, which has been reported for pure polysaccharide and mixed-in calcium phosphate systems before [45,46,47], but not for composites with in situ precipitated calcium phosphates. However, larger pores in the range of several hundred micrometer are needed in order to promote osteogenesis [48], which is why further testing was done only with lyophilized scaffolds.

#### 2.1.4. Shrinking and Swelling

**Figure 4:**
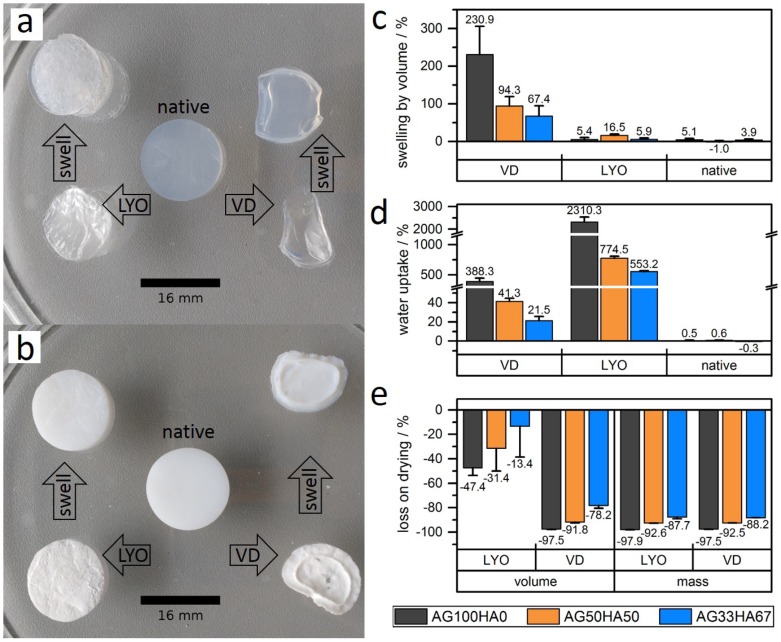
Images of (**a**) agarose hydrogel (AG100HA0) and (**b**) composite hydrogel (AG33HA67) in the native state, lyophilized (LYO), or vacuum dried (VD) and after reswelling of the dried gels. Comparison of (**c**) swelling, (**d**) water uptake, and (**e**) volume and mass loss for dried and native gels.

Shrinking and swelling of the scaffold are important characteristics, as they influence the mechanical properties, drug release, and handling after preparation [32]. Shrinking behavior was tested with drying at 40 ${}^{\circ }C$ under vacuum (VD) or lyophilization (LYO). Upon vacuum drying, the original geometry collapsed completely regardless of the composition (Figure 4a,b); only 2.5–21% of the original volume remained (Figure 4e). Lyophilization, however, resulted in increased form stability (Figure 4a,b), with 52% (AG100HA0), 69% (AG50HA50), and 85% (AG33HA67) of the respective original volume remaining (Figure 4e). Both drying methods removed most of the water from the hydrogels, which was indicated by the mass loss (Figure 4e). AG100HA0, a hydrogel with 2%wt polymer, lost about 98% of its mass, AG50HA50 (2%wt AG, 2%wt HA, and ca. 3%wt NaNO_3_ in the unwashed hydrogel) about 93% of its mass, and AG33HA67 (2%wt AG, 4%wt HA, and ca. 6%wt NaNO_3_ in the unwashed hydrogel about 88 of its mass.

**Figure A2:**
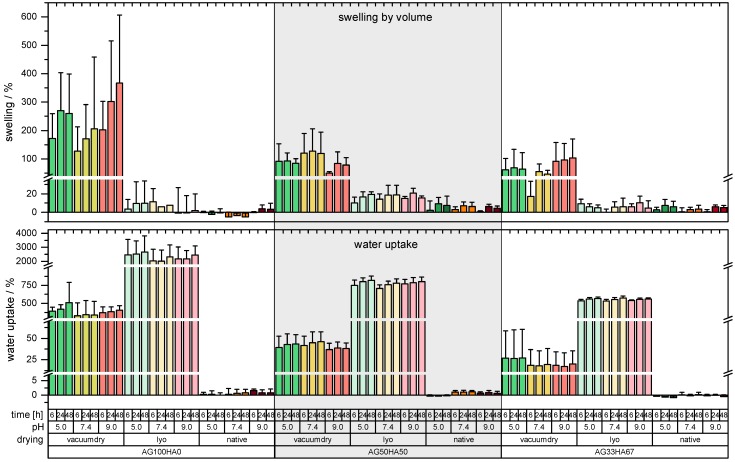
Complete dataset of swelling by volume and water uptake experiments. All three scaffolds were tested at different pH (5.0, 7.4, and 9.0) over 48 h after different drying methods (vacuum drying, lyophilization, and as native hydrogel).

Swelling tests of dried gels and native hydrogels at different pH (5.0, 7.4, and 9.0) for up to 48 h showed no pH-dependency for any sample. Additionally, there were no differences between measurements after 6 h, 24 h, and 48 h, indicating a fast re-swelling of dried samples (see complete data in Figure A2). Native (undried) hydrogels exhibited almost no swelling (not more than 5%), as they took up less than 1% of water (Figure 4c,d). Vacuum-dried samples had the highest swelling ranging from 230% for AG100HA0 to 68% for AG33HA67 (Figure 4c). The water uptake for AG100HA0 (388%) was almost 10-times higher than for AG50HA50 (41%) and even lower for AG33HA67 (22%). The good form stability visually observed on lyophilized samples was confirmed by a very low swelling in volume (less than 17% for all samples), but a very high uptake of water (2310% for AG100HA0). Again, less water uptake was observed for samples with a higher hydroxyapatite content (775% and 553%, respectively) (Figure 4d).

#### 2.1.5. Mechanical Properties

**Figure 5:**
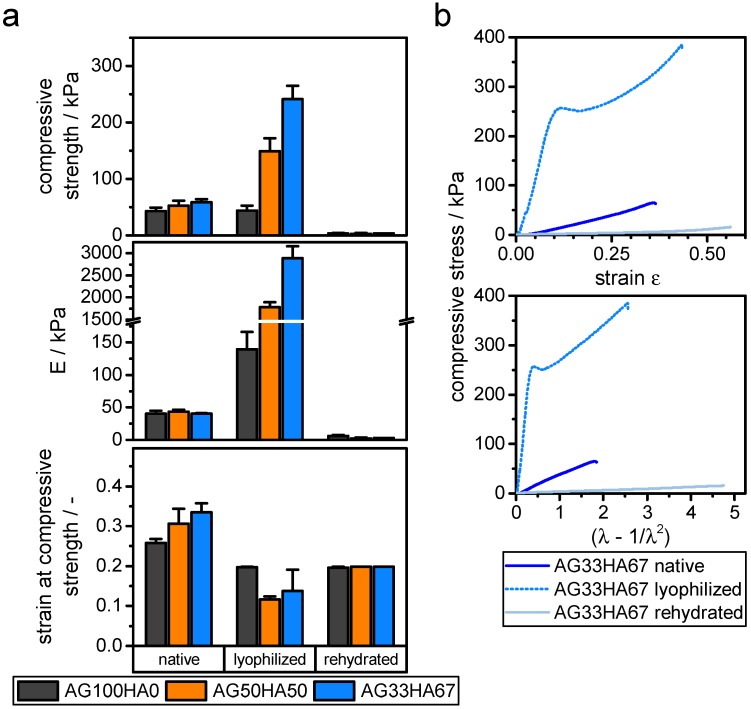
Compressive properties of native hydrogels, lyophilized, and rehydrated gels. (**a**) Compressive strength, elastic modulus *E*, and strain at compressive strength, derived from uni-axial unconfined compression (n=6). (**b**) Typical stress-strain and stress ($\lambda -1/\lambda^{2}$) curves for AG33HA67 gels.

Mechanical compression testing revealed a hyperelastic behavior for native and rehydrated hydrogels. Lyophilized gels followed a linear elastic behavior under strains below 15%. Compressive strength was derived from the first maximum of the strain-stress curve, where the respective strain was also noted. Elastic moduli for lyophilized samples were derived from Hooke’s law in the range below 10% strain. For hyperelastic samples, the behavior could be better approximated with a neo-Hooke model, giving a linear dependency in the stress ($\lambda -1/\lambda^{2}$) curves with $\lambda =L/L_{0}$ being the stretch ratio (Figure 5) [49]. Elastic moduli as a measure for stiffness ranged around 40–50 kPa for native gels, which is a typical value for hydrogels [49,50]. After lyophilization, stiffness increased drastically with increasing amounts of hydroxyapatite, ranging from 140 kPa for AG100HA0 up to 2880 kPa for AG33HA67. Rehydrated cryogels lost their stability, yielding compressive moduli around 2–6 kPa and thus ranging even below the native hydrogels (Figure 5). The composition of hydrogels did not have a significant influence on compressive strength and strain at that stress. All native gels were around 50 kPa and 30% strain, while rehydrated gels were around 3 kPa and 20% strain. Lyophilized gels had higher compressive strengths with increasing amount of hydroxyapatite; however, the strain at that stress did not change significantly.

Literature data of such systems are very vague, because many groups used different approaches to measure stress, strain, and modulus. For a 2% agarose hydrogel, a Young’s modulus of 28 kPa using a neo-Hooke model has been reported [50], while other different groups reached elastic moduli between 2 and 300 kPa for 2% agarose gels [49]. For a comparable composite, elastic moduli for a system of 40% agarose and 60% calcium phosphate measured at 10%, 25%, and 50% strain of 8.6, 24.8, and 60.9 MPa, respectively, have been published [30]. In contrast, the moduli presented in the current study were calculated between 2 and 10% strain and showed a different stress-strain curve, which led to these lower values. These mechanical properties, together with shrinking and swelling, have some implications on practical use. On the one side, scaffolds that have been lyophilized and rehydrated prior to implanting might not be suitable for any major load-bearing applications. However, they might be useful for filling voids and provide structural guidance for cells. Cells could easily migrate into scaffolds with larger pores, which could result in a better vascularization of the scaffold [51,52].

### 2.2. Biocompatibility

**Figure 6:**
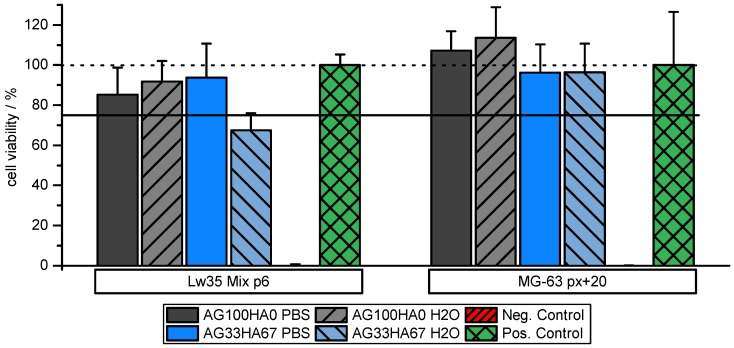
Cell viability of hMSCs Lw35and MG-63 cell lines on pure agarose (AG100HA0) and AG33HA67 composites. Scaffolds washed with PBS prior to MTT assay display higher cell viability. One hundred percent viability (dotted line) and 75% viability (solid line) are marked for clarification.

In order to investigate the biocompatibility of the scaffold materials, MTT assays with scaffold-incubated media were performed on an osteosarcoma cell line and on primary hMSCs. Overall, MTT assays on hMSC (Lw35) and the osteosarcoma MG-63 cell line showed no toxic effect of the scaffolds. Cell viability of more than 95% for MG-63 cells showed that they were unaffected by all types of scaffold regardless of their composition. However, as expected, hMSC seemed to be slightly more sensitive to scaffold material. While they were rather unaffected by the pure AG scaffolds maintaining a cell viability of more than 85%, AG33HA67 washed with water slightly decreased cell viability to around 70%. Washing these scaffolds with PBS instead of water could retain a high cell viability of around 95% (Figure 6). This might be related to the physiological salt concentration of PBS and thus a lower gradient upon scaffold reconstitution. These results show that the materials are non-cytotoxic and are suitable for use in cell culture.

### 2.3. Release Studies

Different release models, namely Weibull, first-order, and Korsmeyer–Peppas, were used in order to gain information on release kinetics. As stated in the Materials and Methods Section, the three models were selected according to their varying amount of information [53]. In the literature, if at all, usually only one fitting model is applied without showing potential differences between them. Often, only a value of how many days or hours until a specific percentage of released drug ($d_{x}$) is given, without any release kinetics parameters. Water-soluble drugs are usually released within several hours [30,38]. ATP and suramin were chosen as model drugs in this study, as they are readily available and show structural resemblance to other P2 receptor ligands that influence osteogenic differentiation [8,11].

**Figure 7:**
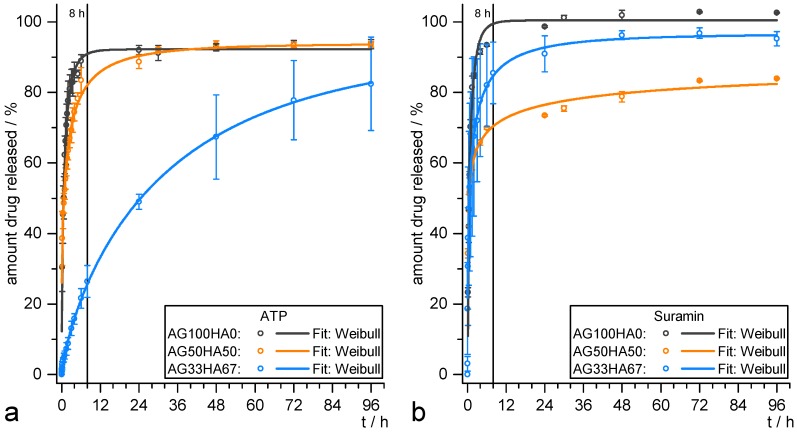
Release data of (**a**) ATP and (**b**) suramin from AG100HA0 (black), AG50HA50 (orange), and AG33HA67 (blue) scaffolds. Data fit: Weibull equation $M=M_{f}\;\cdot \;\left(1-e^{\frac{-{\left(t-T\right)}^{b}}{a}}\right)$.

The release profiles for ATP and suramin are depicted in Figure 7a,b, respectively. They all showed a burst release, where a major amount of the incorporated drug was released within the first 8 h, which is a typical behavior for hydrogel systems and the release of hydrophilic drugs into aqueous media in general. For ATP, a higher amount of HA resulted in a slower release, visible in the decreased slope. Suramin release seemed to be less dependent on the material, and remarkably, the AG50HA50 scaffolds showed the lowest overall amount released after 96 h. Table 2 summarizes the relevant parameters for the different fitting functions of both ATP and suramin release profiles. They can all be fitted with a Weibull release function with $R^{2}>0.993$ accurately describing the amount of drug *M* at time *t*. In the case of ATP, all systems released up to about 92% of the drug, and the latency time *T* was zero (within the accuracy). However, this function was not based on kinetic considerations and thus only useful for direct comparison of the different profiles for which the parameter *q* was used. A high value of *q* represents a slower release. It increased with increasing amount of HA in the material from 0.83 to 34 h, indicating a slower release of the drug. This can also be derived from the decreasing value of release rate *k* in the first-order model, while $M_{f}$, however, represents the release profiles especially up to about 85% release, but usually underestimates the maximum amount released, as it di for all three samples. In the Korsmeyer–Peppas model, the exponent *n* is related to the release mechanism. For cylindrical systems, the Fickian model (Case I) would be at n=0.45, when the release is solely governed by diffusion. With 0.45<n<0.89, there is anomalous transport behavior, where diffusion and system relaxation processes occur. After that, Case II (n=0.89) and Super Case II (n>0.89) models apply. The Korsmeyer–Peppas model reveals a diffusion-based mechanism for AG100HA0, while the exponents of the other samples indicate a more complex release mechanism. The low nof 0.24 for AG50HA50 may be due to physico-chemical interactions between drug and scaffold, while the n of 0.64 for AG33HA67 suggests an anomalous transport, sustaining the release.

In the case of suramin, none of the samples exhibited clearly sustained behavior. The Weibull model calculated an overall amount released of 90–100% with the latency times *T* being slightly increased for AG100HA0 and AG50HA50 and being around zero for AG33HA67. Although AG50HA50 had the lowest overall release, AG33HA67 exhibited the slowest release (q=1.19 h for AG33HA67 vs. q=0.9 h for AG50HA50). This was also confirmed by the first-order release rate k=1.4 h^−1^ and k=0.9 h^−1^ for AG50HA50 and AG33HA67, respectively. The Korsmeyer–Peppas model revealed mainly anomalous transport mechanisms for AG100HA0 and AG33HA67, and again, a low n of 0.30 for AG50HA50, indicating drug-scaffold interactions.

These results showed that both drugs were released more slowly from the system when HA was added to the scaffold. For ATP, the effect was more distinctive, which might be due to a high affinity of the ATP’s triphosphate group to the scaffold’s calcium ions. Suramin was less retained, although a slight decrease in the release rate was also noticed, which could be attributed to the sulfonate–calcium interaction, although the calcium sulfonates remained water-soluble [54]. Although both drugs are highly water-soluble, the release can be slowed to several days, which is longer than comparable systems previously reported in the literature [30]. On the one hand, the burst release behavior could be beneficial for the incorporation of anti-inflammatory or antibacterial drugs, in order to achieve a fast therapeutic effect. A prolonged release on the other hand is needed for drugs that should guide directed stem cell differentiation and need to be on site over a longer period of time.

## 3. Materials and Methods

Agarose (Roti^®^garose, Standard) was purchased from Roth, Karlsruhe, Germany. Phosphate-buffered saline (PBS), Dulbecco’s Modified Eagle Medium (DMEM), fetal bovine serum (FBS), and penicillin/streptomycin were all purchased from BioChrom GmbH, Berlin, Germany. 3-(4,5-Dimethylthiazol-2-yl)-2,5-diphenyltetrazolium bromide (MTT) was purchased from AppChem, Darmstadt, Germany. All other chemicals were of analytical grade and used as received from Merck, Darmstadt, Germany. The same batches of materials were used throughout the study.

### 3.1. Preparation of Hydroxyapatite

Aqueous solutions of NH_4_(HPO_4_)_2_ (1 mol L^−1^, 50 mL) and NaOH (1 mol L^−1^, 64 mL) were added dropwise to 50 mL of an aqueous solution of Ca(NO_3_)_2_ (1.6 mol L^−1^) under stirring at 1000 rpm at 40 ${}^{\circ }C$ within 2 h. Afterwards, the precipitated HA was stirred for an additional 22 h before washing with distilled water and collecting the precipitate by means of vacuum-filtration and subsequent lyophilization (Alpha 2-4LD plus, Christ, Osterode, Germany, −82 ${}^{\circ }C$, 0.1 mbar). The obtained powder was then treated in an oven (M104, Kendro Laboratory Products GmbH, Langenselbold, Germany) at 500 ${}^{\circ }C$ for 2 h.

### 3.2. Preparation of Agarose Gels

Agarose (0.4 g) was dissolved in 20 mL Millipore water at 90 ${}^{\circ }C$. After cooling to around 60 ${}^{\circ }C$, the solution was cast into cylindrical molds (16 mm diameter) and was allowed to cool down, solidifying in the process. The gels were cut into 10 mm high cylinders and subsequently used as hydrogels or dried via lyophilization or supercritical drying. For lyophilization, samples were frozen at −40 ${}^{\circ }C$ and then dried at −82 ${}^{\circ }C$, 0.1 mbar. For supercritical drying, water was gradually replaced with ethanol in multiple steps, before being subjected to supercritical CO_2_.

### 3.3. Preparation of AG/HA Composites

Agarose (0.6 g) was dissolved in 20 mL of a 0.2 mol L^−1^ Ca(NO_3_)_2_ solution at 90 ${}^{\circ }C$. After cooling to 60–70 ${}^{\circ }C$, and 10 mL of 0.12 mol L^−1^ NH_4_(HPO_4_)_2_ and 0.16 mol L^−1^ NaOH were added dropwise over 1 h. The slurry was stirred at 600 rpm for an additional 4 h at that temperature before casting the gels as described in Section 3.2. The cooled gels were then cut and dried similarly to the agarose gels. Scaffolds were prepared in the following polymer/ceramic mass ratios: AG100HA0 (2%wt agarose in hydrogel), AG50HA50 (2%wt agarose, 2%wt hydroxyapatite in hydrogel), and AG33HA67 (2%wt agarose and 4%wt hydroxyapatite in hydrogel).

### 3.4. Characterization of Scaffolds

X-ray powder diffractograms were recorded with a D2 PHASER Diffractometer (Bruker AXS, Karlsruhe, Germany) with a 300 W X-ray tube (Cu Kα, 2θ: 15–65${}^{\circ }$). Samples were dried, pulverized, and analyzed independently. The diffractograms were compared with JCPDS PDF No. 09-432 for HA identification. Crystallite size has been evaluated using the Scherrer equation with the full width at half maximum of (002) signal, as measured with OriginPro 2016. The degree of crystallinity (X_c_) was calculated according to the literature by comparing the intensity of the valley between (112) and (300) V_112/300_ (Equation (1)) to the intensity of (300) I_300_ [55]:

(1) $$X_{c}\approx 1-\left(\frac{V_{112/300}}{I_{300}}\right)$$

Fourier Transform-Infrared (FT-IR) measurements were performed with a FT/IR-410 spectrometer (Jasco, Tokyo, Japan) in absorbance mode with a resolution of 2 cm^−1^. Powdered samples were mixed with dried KBr and subsequently pressed into discs containing approximately 1%wt of sample. Each spectrum was the result of 16 averaged scans.

A LEO 1450 VP scanning electron microscope by Zeiss, Oberkochen, Germany, with a resolution of 4 nm was used for recording micrographs of gold-sputtered samples after lyophilization or supercritical drying. For pore size evaluation, ImageJ software was used on the micrographs. A Bruker XFlash^®^ 6|60 QUANTAX EDS system (Bruker Nano GmbH, Berlin, Germany) was used for electron dispersive X-ray spectroscopy (EDX) analysis at 15 kV.

A Monosorb^TM^ (Quantachrome Instruments, Boyton Beach, FL, USA) BET gas ad-/de-sorption device (Brunauer–Emmett–Teller) with test gas He + 30 N_2_ and adsorption with liquid N_2_ at 77 K was used on both lyophilized and supercritically-dried samples. Analysis was performed in triplicate with approximately 15 mg of sample in each measurement. One-way ANOVA with the post hoc Tukey test with 95% significance was performed on the data using OriginPro 2016 (OriginLab, Northampton, MA, USA).

Shrinking (by mass and volume) during drying was determined by taking the height, diameter, and weight of the native hydrogels and after drying using a caliper.

(2) $$\mathrm{mass}\;\mathrm{loss}\left(\%\right)=\frac{m_{wet}-m_{dry}}{m_{wet}}$$

(3) $$\mathrm{volume}\;\mathrm{loss}\left(\%\right)=\frac{V_{wet}-V_{dry}}{V_{wet}}$$

Similarly, swelling behavior (by volume) and water uptake of the hydrogels and dried samples were investigated by immersing the specimens in 20 mL of water (pH 5.0 and 9.0) or phosphate-buffered saline (PBS, pH 7.4) at room temperature. The swelling was measured by taking the height, diameter, and weight of the scaffolds before immersion and after defined intervals of time, wiping off the excess water with filter paper [56].

(4) $$\mathrm{water}\;\mathrm{uptake}\left(\%\right)=\frac{|m_{dry}-m_{wet}|}{m_{dry}}$$

(5) $$\mathrm{swelling}\left(\%\right)=\frac{|V_{dry}-V_{wet}|}{V_{dry}}$$

A Zwick/Roell universal testing machine Z020 (Zwick/Roell, Ulm, Germany) with an AST 500 N load cell (0.03 N pre-load, crosshead speed 1 mm min^−1^) was used for unconfined uniaxial compression testing. Specimen were cut into approximately 10 mm-high cylinders (exact height measured during analysis) and measured in triplicate at ambient conditions. The compression modulus was calculated after a Hookean model for lyophilized samples and a neo-Hookean model for hydrogels and rehydrated samples, respectively [49].

### 3.5. Biocompatibility Testing

One batch of AG100HA0 and AG33HA67 scaffolds was prepared for biocompatibility testing as followed: After gelation, gels were washed three times with 50 mL deionized water and two times with 50 mL ethanol:water (70:30) for solvent exchange and disinfection. Gels were then cut into small discs of 1–2 mm in height and 12 mm in diameter. Three of each were placed in sterile distilled water or PBS (pH 7.4) for 48 h before lyophilization. Lyophilized scaffolds were incubated in triplicate for 76 h at 37 ${}^{\circ }C$, 5 CO_2_ in a medium comprised of DMEM + 10% FBS + 100 U mL^−1^/100 μg mL^−1^ penicillin/streptomycin. After incubation, the medium was collected and used for the MTT assay. Human mesenchymal stem cells (hMSCs) from adipose tissue of a 35-year old female donor (Lw35) and cells of the osteosarcoma cell line MG-63 were seeded into a 96-well plates at 20,000 and 50,000 cells/well, respectively and were incubated with scaffold-incubated medium for 16 h. Cells were then washed with PBS and cultivated with DMEM + additives (see above) + 0.5 mg/mL MTT for 4 h. Living cells convert MTT into its insoluble formazan, indicated by a color change from yellow to violet. DMSO was used for cell lysis and dissolution of formazan crystals. Cell viability was measured with a photometer (Anthos 2010, Biochrom Ltd., Cambridge, UK) at 550 nm.

### 3.6. Drug Loading and Drug Release Test

Lyophilized cylindrical scaffolds (approximately 10 mm high, 16 mm in diameter) were loaded with drug solution (aqueous ATP or suramin) using the “drop-in” technique of a known amount of drug and equilibrated for several hours at room temperature to allow homogeneous drug distribution in the scaffold. After that, scaffolds were lyophilized and stored at 2–8 ${}^{\circ }C$. For release testing, three of each loaded scaffold were separately placed into 20 mL of water. At given time points, 500 μL of release medium were collected and restocked with 500 μL of water in order to maintain a constant release volume. Absorbance of the sample solution was measured via UV-Vis spectroscopy (UV-1650PC, Shimadzu, Duisburg, Germany) at the absorbance maxima λ = 255 nm and 313 nm for ATP and suramin, respectively. The release was monitored up to 96 h, taking into account the restocking of release medium. All release curves were fitted to different models in order to evaluate the underlying mechanism. A Weibull release function was used for accurately describing the amount of drug *M* released at time *t*:

(6) $$M=M_{f}\;\cdot \;\left(1-e^{\left(\frac{-{\left(t-T\right)}^{b}}{a}\right)}\right)\;\mathrm{and}\;\sqrt[{b}]{a}=q$$

$M_{f}$ is the maximum amount of drug released; *T* describes the latency time of the system, which is often (close to) zero; while *a* represents a scaling parameter and *b* the curve’s form, respectively. In order to evaluate the kinetic parameters of release, usually a power law-based Korsmeyer–Peppas model or a first-order-exponential model is used. The latter is used as a straightforward approach to model release kinetics under sink conditions, which is given for all release studies presented.

(7) $$M=M_{f}-B\;\cdot \;e^{-kt}$$

$M_{f}$ again describes the maximum amount released, $B=(M_{f}-M_{0})$, with $M_{0}$ as the initial amount at t=0, ideally being zero, while *k* is the release rate. The Korsmeyer–Peppas model can be used for estimating kinetic parameters by investigating the first 60% of the release curve:

(8) $$M=K\;\cdot \;t^{n}$$

where *K* is the release velocity constant and *n* is the exponent of release, which is related to the release mechanism [53].

## 4. Conclusions

The present study showed a fast, facile, and cost-effective way to produce organic-inorganic biocomposites comprised of agarose and hydroxyapatite suitable for tissue engineering. The structure and hence the scaffold’s properties can be tuned by varying the polymer/ceramic ratio and by different drying methods. A higher amount of hydroxyapatite improved stiffness against compressive force and formed stability upon drying and re-swelling. Composite scaffolds retained biocompatibility and showed no cytotoxic effect against both osteosarcoma MG-63 and hMSC (Lw35) cell lines. The influence of the drying method resulting in a preserved hydrogel network structure in supercritically-dried samples has interesting implications for tuning porosity in future studies. Small pores of just a few nanometers may be essential for successful vascularization, while larger pores facilitate cell in-growth and osteogenesis. Scaffolds were loaded with either ATP or suramin for drug release and exhibited initial burst release, but in the case of the composite, drug release slowed down for both water-soluble drugs and was sustained over four days. Further studies regarding the scaffold–drug interaction with a special focus on sustaining the drug release even longer and the investigation of other drugs, as well as scaffold degradation studies are still needed. With a specifically-tuned release profile, these scaffold systems may become potential candidates for bone tissue engineering and drug delivery applications. 

## Figures and Tables

**Table 1:** Specific surface area measured by BET of lyophilized (LYO) and supercritically-dried (SCD) samples. ${}^{*}$: significant difference at the 95% level ($p<5\times 10^{-6}$) against both composites, ${}^{**}$: significant difference at the 95% level (p=0.00161) against pure agarose.

	AG100HA0		AG50HA50		AG33HA67	
	LYO	SCD ${}^{*}$	LYO	SCD	LYO ${}^{**}$	SCD
$A_{sp}\;\left(m^{2}g^{-1}\right)$	24±1	144±9	28±2	101±1	32±4	98±1

**Table 2:** Release profile fitting parameters for Weibull, first-order, and Korsmeyer–Peppas equations of ATP and suramin released from different scaffolds. n=3.

Drug	Material	Model		
		Weibull	First-Order	Korsmeyer–Peppas
ATP	AG100HA0	$R^{2}=0.9962$	$R^{2}=0.9808$	$R^{2}=0.9734$
		$M_{f}=0.923\pm 0.007$	$M_{f}=0.90\pm 0.02$	$K=0.61\pm 0.02\;h^{-n}$
		T=0.06±0.06 h	B=0.84±0.03	n=0.50±0.07
		q=0.83±0.08 h	k=1.08±0.08 h^−1^	
	AG50HA50	$R^{2}=0.9951$	$R^{2}=0.9143$	$R^{2}=0.9912$
		$M_{f}=0.94\pm 0.02$	$M_{f}=0.90\pm 0.03$	$K=0.532\pm 0.003\;h^{-n}$
		T=0.00±0.09 h	B=0.69±0.06	n=0.24±0.02
		q=1.4±0.2 h	k=0.48±0.09 h^−1^	
	AG33HA67	$R^{2}=0.9997$	$R^{2}=0.9964$	$R^{2}=0.9959$
		$M_{f}=0.92\pm 0.02$	$M_{f}=0.82\pm 0.02$	$K=0.064\pm 0.003\;h^{-n}$
		T=−0.04±0.04 h	B=0.80±0.02	n=0.64±0.02
		q=34±2 h	k=0.040±0.003 h^−1^	
suramin	AG100HA0	$R^{2}=0.9931$	$R^{2}=0.9902$	$R^{2}=0.9989$
		$M_{f}=1.00\pm 0.02$	$M_{f}=0.99\pm 0.02$	$K=0.706\pm 0.007\;h^{-n}$
		T=0.17±0.05 h	B=0.99±0.04	n=0.78±0.03
		q=0.72±0.07 h	k=1.12±0.08 h^−1^	
	AG50HA50	$R^{2}=0.9941$	$R^{2}=0.9176$	$R^{2}=0.9723$
		$M_{f}=0.90\pm 0.08$	$M_{f}=0.76\pm 0.03$	$K=0.56\pm 0.01\;h^{-n}$
		T=0.23±0.02 h	B=0.70±0.07	n=0.30±0.04
		q=0.9±0.7 h	k=1.4±0.3 h^−1^	
	AG33HA67	$R^{2}=0.9987$	$R^{2}=0.9396$	$R^{2}=0.9501$
		$M_{f}=0.966\pm 0.009$	$M_{f}=0.89\pm 0.03$	$K=0.64\pm 0.06\;h^{-n}$
		T=0.060±0.006 h	B=0.77±0.05	n=0.465±0.08
		q=1.19±0.09 h	k=0.9±0.2 h^−1^	

## Abbreviations

The following abbreviations are used in this manuscript:

ATP	adenosine 5′-triphosphate
BET	Brunauer–Emmett–Teller surface analysis
EDX	electron dispersive X-ray spectroscopy
HA	hydroxyapatite
hMSC	human mesenchymal stem cells
LYO	lyophilization
PBS	phosphate-buffered saline
SCD	supercritical drying
SEM	scanning electron microscopy
VD	vacuum dried
